# Interstitial Gingivitis as a Prominent Obvious Early Symptom of Autointoxication and Drug Poisoning

**Published:** 1901-11

**Authors:** Eugene S. Talbot

**Affiliations:** Chicago


### INTERSTITIAL GINGIVITIS AS A PROMINENT OBVIOUS EARLY SYMPTOM OF AUTOINTOXICATION AND DRUG POISONING.[1]

BY EUGENE S. TALBOT, M.D., CHICAGO.

As autointoxication implies self-poisoning, it logically follows that if any excretory organ fails to perform its function the other excretory organs must do the work of that organ. This is accomplished but very imperfectly. The sweat-glands perform their function normally in the summer, but with the first breath of cool weather the glands contract and the liver and kidneys are forced to perform the work of the skin. Autointoxication takes place. The skin of the fingers begins to peel and itching with eruption results. Faulty elimination from the kidneys without disease of the organs will cause cardiac palpitation, headache, mental depression, dermatoses, rheumatism, gout, hysteria, epileptiform and

[1] Chicago Medical Society, February 13, 1901.

exhaustional psychoses. Asthma, hay-fever, adhesion of the lungs from pneumonia, undeveloped lungs and chest walls will cause blood impurities and skin eruptions from want of proper oxygenation of the blood. Faulty action of the liver, followed by constipation, results in sick headache, neurasthenia, drowsiness, skin eruptions, etc.

Dr. J. H. Salisbury [1] includes under autointoxication all diseases and changes in the system by which poisons resulting therefrom are not eliminated.

The more marked forms of autointoxication due to disease of the liver, bowels, kidneys, skin, and lungs, and the poisons from drugs, such as mercury, lead, brass, potassium iodide, bromide, etc., are more obviously existent. In health, autointoxication is never noticed until after the periods of growth are complete. Foods taken into the system are appropriated up to this period. After the tissues have obtained their normal development, although the same quantity of food is taken, so much is not required by the tissues. The waste material is carried into the blood. The amount of food required depends upon waste and repair. This depends to a great extent upon the avocation of the person. The older the person, the more effete matter needs removal. The excretory organs are unable to do the work they did at thirty. The effete matter becomes a poison in the blood.

The alveolar process is not present at birth. It does not commence to form until the teeth begin to appear through the gums. It remains while the temporary teeth are present. When these teeth are lost the process absorbs, but it reappears on eruption of the permanent teeth. When these teeth are lost, the process is again absorbed. The alveolar process simply holds the teeth in place while they are being used for mastication. The process is made up of cancellated bone tissue. Owing to the office it fulfils, it is easily absorbed. It is, therefore, the most transitory and the most easily affected of any structure in the body. It might also be termed a terminal structure, because the arteries and nerves terminate in the bone and gum tissue.

The mouth is a sensitive organ and rapidly indicates disease. Children with stomach and bowel disturbances reflect such lesions

---

[1] Constitutional Treatment of Interstitial Gingivitis, Journal of the American Medical Association, 1900.

on the gums and alveolar process. The tongue changes in color and the lips become black.

Absorption of the alveolar process is an inflammatory process. I have entitled this inflammation interstitial gingivitis. In interstitial gingivitis, therefore, the alveolar process as well as the gum tissue is involved. The inflammation, being interstitial in character, may be brought about by two methods, local and constitutional. Modern dentistry is doing most to produce local irritations resulting in predisposing causes; the application of the rubber dam, clamps, wedging of teeth, correcting irregularities, sharp edges of decayed or filled teeth, crown- and bridge-work, artificial teeth, more particularly ill-fitting plates, over-stimulation in the use of toothpicks, injuries, tartar, accumulation and decomposition of food, tobacco, and everything of a foreign nature that will produce irritation.

The local causes, which are easily recognized and can be handled only by a dentist, do not require discussion at this time.

The constitutional causes of interstitial gingivitis are autointoxication and drug poisoning. The autointoxication from pregnancy and change in climate have a most marked effect upon the alveolar process. The effect of changes of climate from a moderate temperature to extreme heat and to extreme cold, as well as high altitude, will produce the same result. This is noticeable in American soldiers in Cuba and the Philippines. The engineers and workmen on the Jungfrau Railway, two thousand six hundred meters above the sea level, suffer most intensely with this disease.

The alveolar process, containing two or more teeth, becomes involved, resulting in acute inflammation throughout. The pain may consist of slight uneasiness ranging to the most severe pain. The teeth loosen and finally drop out.

Interstitial gingivitis may affect the alveolar process and gums of defective, degenerate, and neuropathic children, children rachitic, or those who have had long illness. They are very susceptible to nervous impulses. People who have obtained their growth, that is, after thirty to thirty-five years of age, are the most susceptible and exhibit the most marked results from autointoxication and drug poisoning.

The poison due to autointoxication and drugs circulates in the capillaries, setting up inflammation. This extends throughout the alveolar process and gums.

Interstitial gingivitis produces four forms of bone absorption, —lacunar, or osteoclast, halisteresis, Volkmann's perforating canal, and osteomalacia, or senile absorption. Halisteresis and Volkmann's perforating canal absorption are naturally most common, since they are due directly to the inflammatory process and are likewise more rapid in their action. Lacunar or osteoclast absorption is nearly always present, but is slow. Osteomalacia or senile absorption is a natural process and attacks every individual sooner or later. Interstitial gingivitis is recognized by puffiness and bleeding of the gums. Absorption of the alveolar process causes a recession of the gums from the necks of the teeth, thus leaving the necks exposed. On account of the transitory nature of the alveolar process, if the inflammatory process be not arrested, the teeth will finally loosen and drop out. Pus infection frequently takes place. This especially occurs about the necks of the teeth, and the resulting products are taken into the stomach, producing indigestion. Treatment consists in the patient's drinking eight or more glasses of pure water each day, in brushing the gums with a stiff tooth-brush three times a day, thereby causing them to bleed, and the employment of proper mouth-washes. Tincture of iodine should be used upon the gums and alveolar process every other day until they are restored to health.

